# Role of Monoamine Oxidase Activity in Alzheimer’s Disease: An Insight into the Therapeutic Potential of Inhibitors

**DOI:** 10.3390/molecules26123724

**Published:** 2021-06-18

**Authors:** Tapan Behl, Dapinder Kaur, Aayush Sehgal, Sukhbir Singh, Neelam Sharma, Gokhan Zengin, Felicia Liana Andronie-Cioara, Mirela Marioara Toma, Simona Bungau, Adrian Gheorghe Bumbu

**Affiliations:** 1 Department of Pharmacology, Chitkara College of Pharmacy, Chitkara University, Rajpura 140401, Punjab, India; dapinderk78@gmail.com (D.K.); aayushsehgal00@gmail.com (A.S.); sukhbir.singh@chitkara.edu.in (S.S.); neelam.mdu@gmail.com (N.S.); 2 Department of Biology, Faculty of Science, Selcuk University Campus, 42130 Konya, Turkey; biyologzengin@gmail.com; 3 Department of Psycho-Neuroscience and Recovery, Faculty of Medicine and Pharmacy, University of Oradea, 410073 Oradea, Romania; felicia_cioara@yahoo.com; 4 Department of Pharmacy, Faculty of Medicine and Pharmacy, University of Oradea, 410028 Oradea, Romania; mire.toma@yahoo.com; 5 Doctoral School of Biomedical Sciences, University of Oradea, 410073 Oradea, Romania; 6 Department of Surgical Disciplines, Faculty of Medicine and Pharmacy, University of Oradea, 410073 Oradea, Romania; abumbu@uoradea.ro

**Keywords:** Alzheimer’s disease, monoamine oxidase, monoamine oxidase inhibitors, monoaminergic neurotransmitters, oxidative stress, neuroinflammation, reactive aldehydes

## Abstract

Despite not being utilized as considerably as other antidepressants in the therapy of depression, the monoamine oxidase inhibitors (MAOIs) proceed to hold a place in neurodegeneration and to have a somewhat broad spectrum in respect of the treatment of neurological and psychiatric conditions. Preclinical and clinical studies on MAOIs have been developing in recent times, especially on account of rousing discoveries manifesting that these drugs possess neuroprotective activities. The altered brain levels of monoamine neurotransmitters due to monoamine oxidase (MAO) are directly associated with various neuropsychiatric conditions like Alzheimer’s disease (AD). Activated MAO induces the amyloid-beta (Aβ) deposition via abnormal cleavage of the amyloid precursor protein (APP). Additionally, activated MAO contributes to the generation of neurofibrillary tangles and cognitive impairment due to neuronal loss. No matter the attention of researchers on the participation of MAOIs in neuroprotection has been on monoamine oxidase-B (MAO-B) inhibitors, there is a developing frame of proof indicating that monoamine oxidase-A (MAO-A) inhibitors may also play a role in neuroprotection. The therapeutic potential of MAOIs alongside the complete understanding of the enzyme’s physiology may lead to the future advancement of these drugs.

## 1. Introduction

Amongst the various neurodegenerative disorders, Alzheimer’s disease (AD), a progressive form of neuronal cell degeneration, is well known to influence older humans and is estimated to affect 131.5 million people by 2050 [1]. AD is the most habitual root of dementia which is characterized by a substantial cognition loss, including intellectual, language, visual-spatial disturbances as well as memory damage [2]. As the disease advances, neuropsychiatric symptoms increase, and daily performance decreases [3]. Pathologically, AD is identified through the deposition of intracellular neurofibrillary tangles (NFTs) and extracellular senile plaques possessing amyloid-β (Aβ) proteins, which, alongside neuronal death and cerebral atrophy, constitutes the hallmark attributes of the disorder. The brain of AD patients is also distinguished via the existence of an “inflammatory” cascade, even in initial phases. Such cascade catalyzes microglia and astroglia activation, which subsequently stimulates multiple signaling pathways [4,5] to generate inflammatory responses like reactive oxygen species (ROS) and cytokines, resulting in oxidative stress [6]. 

Hallmarks of oxidative stress are perceived initially in AD, indicating that ROS could engage in the torrent of episodes producing neurodegeneration [7]. Monoamine oxidase (MAO), an enzyme confined to the superficial membrane of mitochondria [8], is crucially involved in the metabolism of monoamine neurotransmitters and other amines as well [9]. MAO catalyzed oxidative deamination generates hydrogen peroxide (H_2_O_2_), a negotiator of oxidative stress. MAO exists in two forms (monoamine oxidase-A (MAO-A) and monoamine oxidase-B (MAO-B)), encoded by two different genes, and having distinct tissue allocation systems and separate substrate specificity. MAO-B, the main isoform located in the brain [10], deactivates neurotransmitters such as dopamine, trace amines such as 2-phenylethylamine (PEA), and possibly other neuro-modulatory amines like polyamines [11]. 

The expression of MAO-B is enhanced in the hippocampus and cerebral cortex of AD brains in comparison to healthy brains [12] and enhanced degree (more than 3-fold) of active MAO-B are located in reactive astrocytes encircling amyloid-β deposits [13]. This overexpression of MAO-B in astrocytes is theorized to catalyze imprudent metabolism of monoamines and enhanced generation of free radicals and hydrogen peroxide (H_2_O_2_) and thus may feasibly advance the neurodegenerative mechanisms occurring in AD [14]. Such a process seems to be a prodromal episode in AD that continues throughout disease advancement [15]. 

Considering the presumed character of MAO-B in AD, inhibition of MAO-B expression could be predicted to decrease oxidative stress and neurodegeneration, hence potentially detaining the disease progression. Additionally, MAO-B inhibition can adjust the level of neuro-modulatory amines that might be advantageous for intellectual indications. In fact, an irreversible selective MAO-B inhibitor selegiline was discovered to possess a positive impact on cognitive functions in AD patients [16]. Nevertheless, the therapeutical approach of selegiline in AD remains contentious due to negligence in clinical trials. In phase 2 of the clinical trial, administration of lazabemide, a potent MAO-B inhibitor, determines a 20–40% depletion in cognitive decline in comparison to controls [17]. Despite these outcomes were evocative of a treatment impact on symptom advancement, the development of lazabemide was seized due to possible toxicity patterns. Hence, the overall portrayal of the review provides a clearer indication of the neuropharmacological significance of targeting monoaminergic systems in neurodegenerative disorders and the need for future research.

## 2. General Physiology of Monoamine Oxidases 

MAO is a mitochondrial-limited enzyme with peak appearance extent in gastrointestinal and neuronal tissues. MAO occurs in two distinct isoforms, namely MAO-A and MAO-B, which indicates substantial structure resemblance but vary in tissue distribution and their substrate-inhibitor identification regions. They induce the oxidative deamination of multiple monoamines, thus being crucially involved in the metabolization of a range of neurotransmitters. The altered cerebral levels of the neurotransmitters are associated with the pathophysiology of various neurodegenerative diseases such as Parkinson’s disease, depression, and Alzheimer’s disease [18]. 

Flavin binding MOA induces the oxidative deamination of monoamine analogs along with neurotransmitters such as adrenaline and noradrenaline, norepinephrine (NE), dopamine, 2-phenylethylamine (PEA), tyramine, exogenous amines like 1-methyl-4-phenyl-1,2,3,6-tetrahydropyridine (MPTP) and serotonin (5-HT), producing neurotoxic molecules like H_2_O_2_, aldehydes, and substituted amines or ammonia. MAOs are a class of flavoenzyme oxidases in which the oxidation uses O_2_ as an electron acceptor molecule to produce hydrogen peroxide under the influence of a catalyst. This catalytic reaction is based on the character of the substrate for both MAO-A and MAO-B isoforms. Both isoforms of MAO share 73% sequence resemblance, but vary mostly in their substrate-inhibitor specificity, and tissue allocation. The substrate selectivity in MAO-A form has large intrinsic amines like epinephrine, serotonin, and norepinephrine, whereas substrate selectivity in MAO-B form has small amines like b-phenylethylamine, benzylamine, and some common amines for both forms include tyramine and dopamine. 

**Figure 1:**
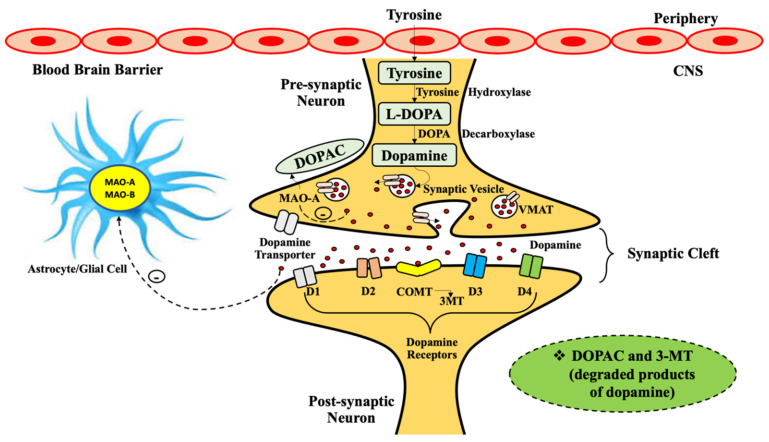
The dopamine synthesis initiates from tyrosine, which crosses the blood–brain barrier (BBB) and transforms into l-dihydroxyphenylalanine (L-DOPA) via tyrosine hydroxylase (TH). Consecutive decarboxylation by DOPA decarboxylase (DDC) transforms L-DOPA into dopamine neurotransmitter inside neurons. Dopamine is entrapped in synaptic vesicles by VMAT-2 (vesicular monoamine transporter 2) or degraded by monoamine oxidase-A (MAO-A) enzyme present in neurons, and by astrocyte and glial MAO-A and MAO-B enzyme into respective degraded products. Dopamine. D1, D2, D3, D4 are dopamine receptors. Legend: L-DOPA—l-dihydroxyphenyl alanine; VMAT—vesicular monoamine transporter; COMT—catechol-O-methyltransferase; 3-MT—3-methoxytyramine; DOPAC-3,4—dihydroxyphenyl acetic acid; CNS—central nervous system.

Mitochondrial-based MAO is mainly located in the human brain, though MAO-A manifests in the intestines, heart, and placenta whereas MAO-B is restricted to cerebral glial cells, platelets, and hepatic cells. MAOs also controls mood, motor activity and assents brain and motivational activities [19,20,21]. In peripheral organs like the intestine, lungs, placenta, and liver, MAOs shield the body by systemic oxidation of amines or prevents their ingression into the blood. It has been observed that MAO-B in capillaries of the blood–brain barrier (BBB) executes a preservative action and acts as a metabolic barricade. It was also acknowledged that intracerebral MAO-A and MAO-B protect neurons against exogenous amines, ceasing the function of amine neurotransmitters, and intrinsic amine store declines in cerebral as well as peripheral tissues. The cerebral monoamines like serotonin, dopamine, and norepinephrine are perpetually metabolized, which is essential for neuronal transmission and to regulate some of the emotional activities (as shown in Figure 1). 

Both isoforms of MAOs, in association with flavin adenine dinucleotide (FAD), induce the oxidative deamination of primary, secondary, and tertiary amines, thus portraying an essential role of FAD in the oxidative deamination of amine substrates. Several neurotransmitters including dopamine, serotonin, and norepinephrine are primary amines that act as electron donors to produce imines and give corresponding aldehydes and ammonia on hydrolysis. 

**Figure 2:**
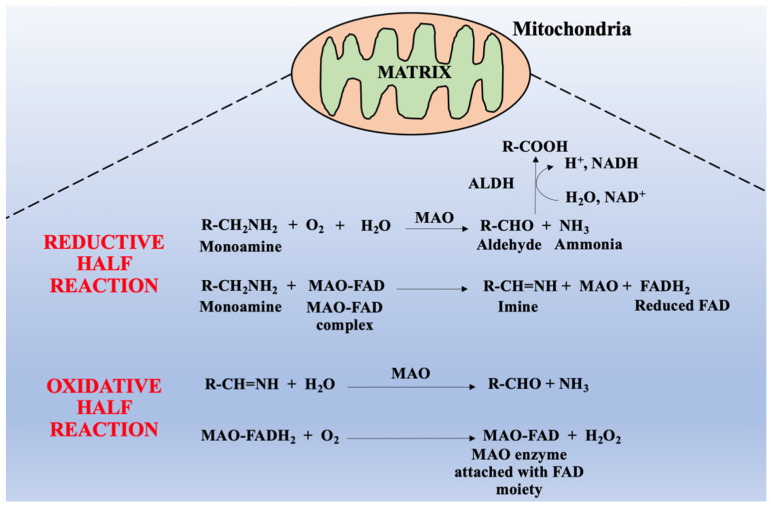
Possible reaction of monoamine-catalyzed generation of reactive species via two successive reactions viz. reductive half-reaction and oxidative half-reaction, leading to oxidative stress. Legend: MAO—monoamine oxidase; FAD—flavin adenine dinucleotide; FADH_2_—reduced flavin adenine dinucleotide; MAO-FAD—monoamine oxidase-flavin adenine dinucleotide complex; H_2_O_2_—hydrogen peroxide; ALDH—aldehyde dehydrogenase; NADH—nicotinamide adenine dinulceotide; NH_3_—ammonia.

The MAO-catalyzed biochemical reaction metabolizes monoamines give respective aldehydes under the activity of aldehyde dehydrogenase (ALDH) enzyme and aldehydes produce acids, alcohols, or glycols by aldehyde reductase (ALR). In these biochemical reactions, various neurotoxic byproducts are produced such as H_2_O_2_ and ammonia (NH_3_). In general, ROS are produced from H_2_O_2_ which is responsible for mitochondrial dysfunction and neuronal death. Generally, this biochemical reaction was further classified into two reactions, half of which is oxidative, and the other half is reductive (as it is shown in Figure 2). 

In the reductive reaction, two hydrogen atoms are transferred to the flavin molecule of MAO-FAD complex from amine substrate, thus reducing flavin adenine dinucleotide (FAD) to reduced flavin adenine dinucleotide (FADH2), while in the oxidative reaction, reduced flavin of MAO-FADH2 complex is oxidized to form FAD (MAO-FAD) which produces H_2_O_2_. Whereas amine substrate is oxidized by elimination of two protons and two electrons, the single-bonded C-N amine substrate is transformed into double-bonded imines [22,23]. The comprehensive pathways of amine oxidation are not debated so far, however, researchers have suggested some amine oxidation processes [24]. Lately, three pathways of MAO-catalyzed oxidation were suggested namely, (1) the hydrogen atom transfer (HAT) mechanism, (2) single-electron transfer (SET) or radical transfer mechanism, and (3) the polar nucleophilic pathway [25]. 

The co-crystal composition of both isoenzymes MAO-A and MAO-B through X-rays with considerable MAOIs has obtained novel understanding into the organization of these enzyme-ligand systems, moreover, it also stimulated the research in the field of MAO blockade as a possible therapeutic regime in neurodegenerative disorders [26]. While electron paramagnetic resonance (EPR) investigations manifested that both isoenzymes are structurally dimeric [27], the crystallographic examinations disclosed that the mammalian MAO-A isoform crystallizes as a monomer [28], though MAO-B crystallizes as a dimer. Moreover, the active site of MAO-A comprises one hydrophobic pocket, whereas the bilateral pocket of MAO-B isoform consists of an entrance hydrophobic site and a substrate-binding site with the FAD cofactor and entrance hydrophobic cavity [29]. 

## 3. Involvement of Monoamine Oxidase in Alzheimer’s Disease

Since the last five decades, MAOIs were clinically utilized as antidepressants. Currently, a multitude of these MAOIs are active in the treatment of neurodegenerative disorders like Parkinson’s disease (PD) and Alzheimer’s disease (AD) [18]. In eukaryotes, MAO is widely distributed in the exterior layer of mitochondria. Altered levels of MAO-A and MAO-B isoenzymes are associated with the degeneration of neurotransmitter biogenic amines such as serotonin, noradrenaline, and dopamine being suggested that they are in relation with multiple conditions such as schizophrenia, depression, antisocial aggressive behaviors, cancer, and neuropsychiatric disorders (NDs) namely PD and AD [30]. 

It was indicated that few symptoms of AD are derived from the variation in dopaminergic, serotoninergic, and monoaminergic neurotransmitter signaling associated with both MAO-A and MAO-B isoforms [31]. The MAO-catalyzed oxidative deamination of amine substrates liberates respective byproducts like ammonia, aldehydes, and hydrogen peroxide may be thus engaged in the advancement of AD. In the brain, the MAO-A isoenzyme largely occurs in catecholaminergic neuronal cells, while the isoform MAO-B is largely located in glia and serotonergic neurons. 

In post-mortem brain biochemical investigations showed that the variations in MAO-A and B in the cortex arise in early phases of AD and remain sustained throughout the development of AD [32]. It has also been noticed that the proportion of MAOs or mRNA is elevated in several cerebral regions such as the parietal, frontal, occipital, and temporal cortex and also in the frontal lobe of the neocortex [12]. This indicates that the mechanism in MAO activity could be transcriptional or post-transcriptional and may be responsible for increasing protein synthesis and is also involved in the progression of AD. As the occurrence of reactive MAO-B in AD brains has been noticed it designates the distinct appearance of MAO-A in several regions of the brains of AD cases. Immunostaining studies revealed that the activity of MAO-B isoform was substantially inclined in the cortical and hippocampal regions of AD brains, demonstrating the primary neuronal loss and significant gliosis in such regions of the brain, whereas MAO-A was raised in the hypothalamus and frontal lobe. 

In patients of AD, the MAO-A expressions seem to be declined in the locus ceruleus, determining 80% neuronal depletion, proposing that reactive MAO-A in neuronal cells are allied in the pathophysiology of AD as a susceptible risk factor [33]. Additionally, inclined MAO-A expression in AD brains appeared more remarkable in glia and the variation in levels of MAO-A expression in AD might play multiple processes [34]. Stimulation of MAO causes cognitive decline in patients with AD. It has been demonstrated that biomolecular monoamine neurotransmitter systems play a substantial character in cognition mainly awareness, paranoid thinking, memory, attitude, and also emotion and behavior [35]. 

Some neurotransmitters (for example: cholinesterase, serotoninergic, glutamatergic, noradrenaline) are distressed by MAO, following cognitive dysfunction. Substantial investigation revealed that MAO-A isoform preferentially controls serotonin and noradrenaline and MAO-B acts positively on benzylamine and 2- phenylethylamine [36]. Noradrenaline plays a crucial role in major activities, ruling intellect, cognition, and motivation that are necessary for social interaction. Activation of MAO is a harmful factor for noradrenaline signaling, impact symptoms of disarranged major activities. Activation of MAO also has a deleterious influence on cholinergic transmission associated with memory and emotion [37,38]. 

Presently, scientists showed notable interconnection between MAO-A and catechol-O-methyltransferase-genotypes, such that accessibility of increased prefrontal catecholamine was associated with ameliorated memory functioning. Although, it is not detailed that there is a direct interrelationship between the MAO and neurotransmitters in AD. Some researchers disclosed that activation of MAO has an indirect connection with several neurotransmitters and cognitive decline in AD. 

**Figure 3:**
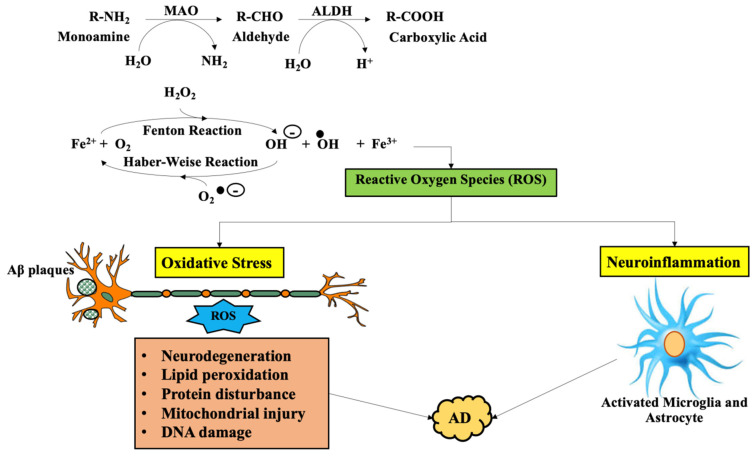
The reactive oxygen species generated by overactive monoamine oxidase in conjunction with Fenton’s and Haber-Weise reaction which successively causes neuroinflammation and oxidative stress; implicated in Alzheimer’s disease (AD). Legend: MAO—monoamine oxidase; ALDH—aldehyde dehydrogenase; NAD^+^—nicotinamide adenine dinucleotide; NADH—reduced nicotinamide adenine dinucleotide; H_2_O_2_—hydrogen peroxide; O_2_—singlet oxygen; ROS—reactive oxygen species; Fe^2+^—ferric ion; AD–Alzheimer’s disease.

In AD, the correlation between MAO activation and oxidative stress is a well-acknowledged source of neurotransmitters impairment, namely, the adrenergic and cholinergic system, which plays a critical role in cognition loss [39]. Additionally, neuroinflammation has a censorious role in cognitive impairment and medium for oxidative stress, while in AD, MAO may act as a proinflammatory cytokine, determining cognitive decline (as it is summarized in Figure 3). Reactive MAO enhances cerebral levels of monoamine and modulates other neurotransmitters, resulting in cogitative loss [40].

In addition, alterations in MAO-linked acid metabolites of serotonin and dopamine (i.e., 5-hydroxyindole-3-acetic acid (5- HIAA) and homovanillic acid (HVA), respectively) are associated with cognitive dysfunction and dementia, as experimentally noticed in several animal models of AD. At first, greater activity of MAO was noticed in brains and platelets of AD cases, though in CNS, activity of MAO-B advances with age as MAO-B level increases. As compared with gender and age, MAO activity was remarkably uplifted in platelets of AD patients, and the MAO-B activity but not MAO-A activity was significantly larger in hippocampus and cortex of the gyrus cinguli of AD patients. Evidence indicated the direct relationship between symptoms, MAO-B activity in platelets and the cerebrospinal fluid (CSF) monoamine metabolites revealed the significance of MAO-B expression in the platelets as the pathological marker of AD. Therefore, enhanced MAO-B expression may account as an indicator for vulnerability towards AD. As per Mini-Mental State Examination (MMSE) of three groups with 23 patients in the early phase, 23 patients in the middle phase, and in 28 patients in the last phase of AD, also matched with 49 elderly controls, the substantial connection between age and MMSE scores and MAO-B activity were recognized, manifesting that such indicators may portray the complexity and therapeutic progression of AD [41]. Activation of MAO-B also contributes to the production of amyloid-β plaques. Observations on the pathophysiological framework of AD have signified that there is an oxidative injury in AD. Oxidative tension in patients of AD catalyzes amyloid-β plaque generation. Enhanced MAO-B activity in hippocampus and cerebral cortex of AD regulates healthy CNS and active MAO-B inclined measure located in sensitive astrocytes all around amyloid-β plaques. In astrocytes, enhanced level of MAO-B, is considered an indicator of oxidative tension, in the imprudent oxidative deamination of monoamines which produces more hydrogen peroxide and free radicals, consequently advances AD progression, such process appears to be an initial stage in the advancement of AD [15]. 

**Figure 4:**
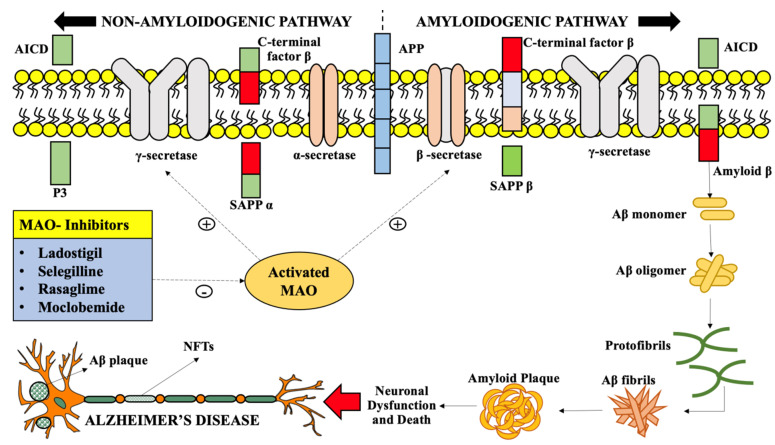
Overactive monoamine oxidase catalyzes the amyloidogenic and non-amyloidogenic cleavage of amyloid precursor protein by directly activating the beta-secretase and gamma-secretase activity, thus leading to aberrant amyloid plaque generation, a hallmark of Alzheimer’s disease. Legend: MAO—monoamine oxidase; NFTs—neurofibrillary tangles; Aβ—amyloid beta; APP—amyloid precursor protein; AICD—amyloid precursor protein intracellular domain; P_3_—amyloid peptide; sAPPα—soluble amyloid precursor protein alpha; C—terminal factor ß–carboxy terminal factor beta; Γ—secretase–gamma secretase; α—secretase–alpha secretase; ß—secretase–beta secretase.

In AD, Figure 4 describes how the monoamine activity may impact the clinical features of a neurodegenerative disorder such as by cleavage of amyloid precursor protein (APP). The molecular biology studies of Aβ production proposed that it results from two consecutive cleavages of APP via two distinct enzymes, namely, ß-secretase (b-site APP cleavage enzyme or BACE) and Γ-secretase following MAO activation [42]. The initial step involves the extracellular ß-secretase cleavage to produce Aβ between APP residues of Met671 and Asp67, generating an insoluble cell membrane-bound segment and an extracellular segment. The cell membrane-bound segment is further cleaved by Γ-secretase in the lipophilic transmembrane domain, subsequently generating Aβ an intracellular fragment of APP. APP generates toxic Aβ fibrils in AD brains under the influence of serotonin via activation of various serotoninergic receptors [43]. Furthermore, acid monoamine metabolites are associated with the cerebrospinal levels of Aβ, while enhanced MAO-B expression in plaques linked with astrocytes is a clinical feature of AD advancement [41]. 

This correlation was currently reassured in imaging 5xFAD using two-photon in the AD model [44]. Lately, MAO-B has progressed as a potential therapeutic target for AD. MAO-B is rigidly associated with the production of Γ-aminobutyric acid (GABA) in susceptible astrocytes, consequently memory loss in an animal model of AD, due to the inhibition of synaptic transmission and declined spike probability by astrocytic GABA, catalyzing impairment of synaptic plasticity. 

Reactive astrocyte-linked generation of GABA is decreased by inhibiting MAO-B, utilizing irreversible MAO-B inhibitors like selegiline (l-deprenyl). Selegiline entirely reinstates cognitive function, impaired spike probability, and synaptic plasticity in AD models, even in the sight of Aβ peptides. This observation was instituted in the treatment of dementia-type AD. Some biomedical published data also detailed that in patients with AD, selegiline recuperates short-term cognition loss, though, long-term treatment is distressing. 

The relationship between oxidative tension and neuroinflammation is a critical feature for the Aβ generation. Additionally, both these factors are crucial in the pathological mechanism of neurodegeneration in AD, signifying that the therapeutic approaches targeting the above 2 processes may be advantageous. It has been described that several MAOIs decrease the Aβ generation by hampering neuroinflammation through inhibiting nuclear factor-kB expression, downregulating the expression of tumor necrosis factor-α and interleukin-1β, diminution of glial stimulation [45]. Therefore, proposals for the progression of designing MAOIs as a ligand drug plays a crucial role in AD treatment. 

Pre-clinical and clinical evidence, suggesting MAO association with progression of Alzheimer’s disease, are presented in Table 1.

## 4. Inhibition of MAO-A and MAO-B as a Therapeutic Approach in Alzheimer’s Disease Treatment

MAOIs raise the monoamine neurotransmitter levels in CNS, namely, dopamine, noradrenaline, and serotonin which were suggested to be practically inadequate in dementia [53]. The MAO-catalyzed reaction induces the generation of reactive aldehydes, H_2_O_2_, and an alkyl-substituted amine or ammonia. Corresponding byproducts are possibly neurotoxic [54], and their generation is hindered by MAOIs. The catalytic oxidation of serotonin and catecholamines by MAO induces the production of 3,4-dihydroxyphenylglycolaldehyde (DOPEGAL) from adrenaline and noradrenaline, 3,4-dihydroxyphenylacetaldehyde (DOPAL) from dopamine, and 5-hydroxyindoleacetaldehyde (5-HIAL) from 5-HT or serotonin. These three metabolites have been proposed to induce neurotoxicity in several pre-clinical and clinical studies [55], thus being involved in the pathophysiology of AD. 

The pathophysiological characteristics of MAOs make a possible progression of MAOIs with therapeutic utilization in the treatment of various disorders like neurodegenerative diseases. MAOIs reduce the repercussions of reactive MAO-B, though they have not been considerably employed as therapeutic approaches in AD treatment; similar researches have been carried out mostly with irreversible MAO-B inhibitors like rasagiline and selegiline, and the outcomes in long-standing researches have been unsatisfied in general [56]. Researchers suggested that the GABA/MAO-B interactivity may account for the improvement in cognitive deficits in AD upon short-term administration of irreversible MAO-B-selective inhibitor, selegiline. 

Studies on the amyloid precursor protein/presenilin 1 (APP/PS1) model of AD, found that long-term administration of selegiline attenuates the abnormal GABA measure, firstly by inhibiting MAO-B, that ultimately upregulates the expression of the compensating GABA-producing enzyme “diamine oxidase”. On the other hand, it was observed that highly selective, reversible MAO-B inhibitors do not show this action and alter cognitive impairment in the AD mouse model. Because of their actions on reactive aldehydes, singlet oxygen species, and primary amine oxidase (PrAO), MAOIs might be functional adjuvant drugs in the treatment of AD, as their GABA-elevating actions may improve memory and learning abilities. All MAO-related enzymes are of potential interest as a therapeutic target in AD due to their high-level expression and potential selectivity. Therefore, selective MAO inhibitors were improved to regulate the MAOs expression. The extensive collection of both selective or nonselective reversible and irreversible MAO inhibitors is now available [57]. MAOIs have been mostly implemented in the treatment of neurodegenerative diseases like AD. 

The combined overview of molecular biology and pharmacology manifests that MAOIs have neuroprotective functions in the treatment of AD. The considerable neuroprotective actions in AD by MAOIs involves: (a) advancement of iron-chelating and anti-oxidant activity thus preventing hyperphosphorylation of tau protein and oxidative stress, respectively, (b) improvement in cognition, and (c) regulation of Aβ and APP gene expression [58,59]. The evolution of multitarget inhibitors with principal MAO inhibiting activity, combined with iron-chelating activity could be successful therapeutic targets in the treatment of neurodegenerative disorders like AD [60]. 

The reason why these inhibitors are CNS specific has not been explicated so far. As mentioned above, it is crucial to produce potent specific MAO inhibitors. Thus, immoderate attentiveness should be concentrated on drug design approaches for the development of potent MAO inhibitors. The following are the three main mechanisms of action of MAOIs, implicated in AD.

### 4.1. Sequestration of Reactive Aldehydes

A salient feature that is of substantial attention in respect of potential neuroprotective functions of MAOIs is their resistance to “aldehyde overload”, that is, an imprudent measure of reactive aldehydes [61]. Reactive aldehydes may cross-link, and form adducts through the formation of a Schiff base, with nucleic acids, proteins, and amino phospholipids, catalyzing several toxic effects. This may have consequences on hindrance in protein, DNA, and RNA synthesis, disturbed calcium equilibrium, disrupted cell membrane permeability, and alteration of pathways regulating cellular respiration [62]. There has been a considerable understanding of the investigation in the past 20 years targeting the entanglement of reactive aldehydes with neurodegeneration [63,64]. Possible origins of these aldehydes involve carbohydrate autoxidation, lipid peroxidation due to oxidative tension, cytochrome P450-induced oxidation of alcohols, myeloperoxidase-induced oxidative deamination of amino acids, and catalytic expression of amine oxidases [65]. 

Aldehyde overload and produced toxicity take place due to decreased metabolism of enzymes like aldehyde dehydrogenase, glutathione-*S*-transferase, and aldo-keto-reductase. Deposition of toxic aldehydes also induces reduction of cellular thiols that sequester the aldehydes, especially the vital antioxidants such as cysteine and glutathione [66]. Clinically, this depletion in intracellular thiols has been observed in AD-related dementia [67]. There have been reports of an increased degree of acrolein and malondialdehyde in the serum, plasma, RBCs, and brains of AD individuals [68,69]. Amounts of 4-hydroxy-2-nonenal (4-HNE) have been reported to be elevated in cerebrospinal fluid (CSF) and brains of AD patients, and this toxic aldehyde has been found in the amyloid plaques and neurofibrillary tangles of AD. 

Raising levels of toxic aldehydes such as 4-HNE and acrolein have also been observed in the brains of patients with early symptoms of AD [70], proposing that an early event in the progression of AD could be deposition of reactive aldehydes. Long-term administration of acrolein to rats has been described to cause mild cognitive impairment together with neuroinflammation and neuronal loss in the hippocampus; some scientists also reported elevated cortical levels of ß-secretase (BACE-1) and demotion in levels of α-secretase in the cortex and hippocampus [71,72].

Additionally, it was observed that long-term exposure to acrolein in mouse models causes learning and memory deficits and that there was a positive association between that deterioration and the CSF concentration of acrolein. Hyperphosphorylation of tau protein and induction of tau agglomeration into fibrils have been observed to be catalyzed by acrolein and methylglyoxal [73]. 

In later stages of AD (i.e., during advanced oxidative stress) an enhanced amount of acrolein, malondialdehyde, and 4-HNE are distinct [74]. MAOIs such as mercapto- and hydroxylamine compounds are well known to “finish off” or sequester toxic reactive aldehydes via a chemical reaction, but some of these drugs are toxic by themselves. Compounds like hydrazine with an unsubstituted NH_2_ moiety also react with toxic aldehydes to form innocuous hydrazones. Phenelzine is one such hydrazine, thus being awaited to be utilized to lower the levels and toxicity of reactive aldehydes. In other research, it was located that phenelzine weakens the depletion in the viability of cultured cortical neurons of mouse models generated by acrolein [75] and mop up various in vitro toxic aldehydes, namely, acrolein, formaldehyde, 4-HNE, and malondialdehyde. 

Song et al. performed exhaustive research on the actions of phenelzine on formaldehyde-mediated damage to in vitro primary cortical neuronal cells and astrocytes. Formaldehyde obstructed glutamate take-up by reducing glutamate transporters expression in astrocytes and stimulated the secondary messenger p38 mitogen-activated protein kinase (p38 MAPK), which takes part in a pathway regulating cellular reactions to stress and cytokines, and these actions were reduced by phenelzine [75]. Phenelzine has also been observed to reduce 4-HNE-mediated mitochondrial damage in an animal model [76] of traumatic brain injury [77] and to alleviate 4-HNE-mediated injury to lipids and proteins in blood [78].

### 4.2. Incline in GABA Levels

Despite the fact that it was used as an MAOI, it is thoroughly reported that phenelzine also induces an increase in CNS levels of GABA in animal models. In 1969 Popov and Matthies observed that the exposure of MAOIs in rats before administering phenelzine, reduced the capability of phenelzine to raise GABA levels in the brain, proposing that a metabolite produced by the action of MAO on phenelzine accounts for the perceived actions. As ß-phenyl ethylidene hydrazine (PEH) had been observed as a metabolite of phenelzine, it was indicated that PEH induced a rapid, distinct, and comparatively persistent advancement of brain GABA levels following a single intraperitoneal (i.e.) injection to mouse [79]. 

Although PEH is a weak MAO inhibitor [80], it inhibits the GABA transaminase (GABA-T) enzyme [81], probably supporting the GABA-raising function of phenelzine. The attentiveness in potential neuroprotective effects of MAOIs was evoked by records implying that several GABAergic agents reduced neurodegeneration in ischemic stroke models [82] and suggesting that such drugs were effective by preventing the detrimental neurotoxic actions of the enhanced glutamate release occurring in ischemic stroke [83]. 

In fact, there is now colossal literature designating the significance of continuing the neuronal equilibrium between glutamate and GABA and proposing that a disturbance in that equilibrium is a characteristic of various neurological and psychiatric disorders, such as epilepsy, multiple sclerosis, mania, schizophrenia, and depression [84,85,86,87,88]. The scrutinization of MAOIs was presented in an ischemic model in the gerbil (an animal model) that located noticeable neuroprotective actions with such doses of these drugs which causes an elevation in levels of GABA in the brain [89,90].

### 4.3. Inhibition of Primary Amine Oxidase

Primary amine oxidase (PrAO) is a copper-linked laminal glycoprotein, and the extracellular part may be split off, forming an ambient configuration in blood plasma. In some organelles, the laminate configuration is similar to Vascular Adhesion Protein (VAP-1) engaged in translocation of white blood cells (WBCs) at inflammatory sites [91,92]. The reactive aldehydes are produced by oxidative deamination of monoamines, through the ambient plasma configuration and the lamina-linked configuration of PrAO. Such aldehydes have manifested to enhance the generation of amyloid-β (Aβ), β-sheets and protofibrils [93], the pair of which are suggested to be a considerable risk to the pathophysiology of AD. Enhanced serum PrAO expression has been observed in patients with AD [94,95,96] being described a robust activity of PrAO colocalized with Aβ plaques on neurons of autopsied brain samples from AD individuals. Raised PrAO plasma expression has also been observed in multiple sclerosis, diabetes patients, and PrAO inhibition has been beneficially described in the experimental autoimmune encephalomyelitis (EAE) model of multiple sclerosis [97] showing anti-inflammatory effects that are advantageous to vascular health [98]. Ischemia-reperfusion damage in an animal model of stroke is reduced in PrAO-deficit mice and through PrAO inhibitors. 

In addition, PrAO inhibition has been observed to contribute to anti-inflammatory protection in a mouse model of intracerebral hemorrhagic stroke [99,100] describing that a PrAO inhibitor had anti-inflammatory and analgesic actions in the chronic arthritis model. It is a concern that MAOIs like phenelzine have also manifested to be a potent PrAO inhibitor; also, it has been indicated that PEH is a strong in vitro inhibitor of PrAO in clinical studies and that intraperitoneal (i.p.) injection of MAOIs enhances brain levels of methylamine, a secondary measure of depletion of reactive aldehyde levels. These studies and the observations related to the aforementioned actions of MAOIs indicate that research on the actions of MAOIs on AD models is justified.

### 4.4. Miscellaneous Neuroprotective Actions of Monoamine Oxidase Inhibitors

The reactive nitrogen species (RNS), peroxy-nitrite is formed after the reaction between nitric oxide and superoxide anion. Peroxy-nitrite is believed to cause toxic actions following its decomposition producing a hydroxyl radical, nitrogen dioxide radical, and a nitryl cation, all of which may result in marked neurodegeneration in AD [101,102]. Peroxy-nitrite has been related to a variety of disorders like hypertension, diabetes and may precipitate aging [103]. Studies outlined that MAOIs are preserved from protein nitration, protein carbonyl production, and lipid peroxidation in peroxy-nitrite-exposed platelets and plasma samples. The researchers deduced that the potential of MAOIs to scavenge toxic aldehydes was in charge of its neuroprotective functions in all three of these oxidative stress conditions. 

Effects of MAOIs on the brain-derived neurotrophic factor (BDNF) could also be responsible for the neuroprotective actions of these drugs. Chronic exposure of various antidepressants together with MAOIs has been observed to catalyze an incline in central BDNF levels of an AD model [104]. In an experimental study, it was discovered that long-term administration of MAOIs results in enhanced mRNA expression of BDNF in the hippocampus and frontal cortex and countermand the corticosterone-induced reduction in the BDNF expression in the cortical regions [105].

The dopamine complexed neurotoxin, 1-methyl-4-phenyl-1,2,3,6-tetrahydropyridine (MPTP) is metabolically transformed into 1-methyl-4-phenylpyridinium (MPP^+^) via MAO-B, which has been suggested to induce neuronal loss by altering the mitochondrial permeability [106]. The effects of MPP^+^ on differentiated PC12 cells were also reported and outlined that MAOIs decrease MMP^+^-catalyzed nuclear fragmentation and reversed the decline in mitochondrial membrane potential, ROS generation, cytochrome C release, exhaustion of total glutathione extents, and neuronal death caused by H_2_O_2_.

The neural cell adhesion molecule L1CAM (L1) performs a crucial function in the growth of the central nervous system and is believed to be associated with multiple neurodegenerative disorders like AD [107]. In spinal cord injury, L1 can induce axonal regeneration and increase remyelination, neuronal survival, and synaptic plasticity [108]. In an experimental study, researchers suggested that MAOIs was an L1-mimetic as it enhances the expression and proteolysis of L1 and phosphorylation of extracellular-signal-regulated-kinase (Erk) caudal to the injured site. In another research, it was observed that MAOIs prevented the toxicity induced by the environmental neurotoxin paraquat- it was indicated that such drugs counteracted the decline in dopamine levels and tyrosine hydroxylase expression, decreased ROS formation, maintained mitochondrial viability, supported the antioxidant system, and averted a reduction in adenosine triphosphate (ATP) levels.

Phosphorylation of Fas-associated death domain (FADD), and death receptor mediator, can cause induction of anti-apoptotic activities [109]. It has been outlined that short-term exposure to MAOIs in animal models determines a noticeable incline in the proportion of phosphorylated to non-phosphorylated FADD in the cortical region, and the researchers proposed that this anti-apoptotic action of MAOIs could be associated with its GABA-raising function, following stimulation of GABA-A receptors.

In metabolomics investigations, it was also indicated that i.p. administration to male rats of MAOI causes a noticeable incline in cortical levels of ornithine and a range of *N*-acetylamino acids. It was reported that the enhanced levels of ornithine may be a marker of reduced production of polyamines or glutamate (consequently reduced generation of reactive aldehydes like acrolein), hence providing neuroprotection. The potential participation of the actions on *N*-acetylated amino acids in neuroprotection requires a further understanding of the role these *N*-acetylated amino acids have in the brain, though the occurrence in minute concentrations in CNS and is an indicator for viable neurons [110].

Table 2 describes the MAO inhibitors used in the preclinical trials for the treatment of Alzheimer’s disease.

## 5. Therapeutic Potential of Monoamine Oxidase Inhibitors in Multiple Neurodegenerative Diseases

A broad variety of monoamine oxidase inhibitors (MAOIs) that comprises irreversible and reversible inhibitors of MAO-A, MAO-B, or both are available now, which prove to have the therapeutic advantage in various disorders such as stroke, neurodegenerative disorders, and aging. The therapeutic potential of MAOIs in distinct neurodegenerative disorders is explained hereunder; MAOIs have been therapeutically used for years in the treatment of depression [115]. The antidepressant effects of MAOIs are due to selective neuronal inhibition of MAO-A, which enhances the levels of dopamine in the CNS. The reversible MAO-A inhibitors have been proved to be specifically potent in the treatment of depression in aged patients [116]. 

Selective MAO-A and non-selective MAOIs came across as therapeutically active in the treatment of phobia and atypical depressions, like those including bulimia, hysterical traits, hypersomnia, tiredness for which they are better than amine-uptake inhibitors. MAO-B inhibitors lack the antidepressant property [117,118] and do not assist the chain reaction except when administered at doses sufficient to inhibit MAO-A. The application of MAOIs as dopamine-sparing drugs or as adjuvants to levodopa was examined for the treatment of Parkinson’s disease (PD), but such an approach with non-selective inhibitors was rejected. MAO-B levels are enhanced in the brains of patients with Parkinson’s disease as a result of gliosis, since the human basal ganglia have elevated MAO-B than MAO-A expression, and because dopamine is uniformly metabolized by both isoenzymes in humans, the selective MAO-B inhibitor selegiline was earliest examined as an adjunct to levodopa. Selegiline is efficacious both as monotherapy and as an adjuvant to levodopa [119]. 

However, selegiline was shown to delay disease advancement during the initial stages of the treatment, no suggestive action on the course of the disease was located after that time [120]. Current indications propose that rasagiline and lazabemide can also slow the progression of Parkinson’s disease [121]. Although, a contemporary study of the available evidence has deduced that there is inadequate data to suggest that any MAO-B inhibitor considerably slows early disease progression and that more study is needed. Initial research on the activity of MAO-B inhibitors on patients with PD was disturbed by the use of insufficient washout periods. Moreover, MAO-B inhibitors can relieve the symptoms of the disease to a certain extent; failure to consider this data might clear the somewhat overenthusiastic explanation of initial clinical results [122]. The pathways underlying these symptoms are not completely acknowledged, but MAO-B inhibition and increased levels of dopamine and 2-phenylethylamine (2-PEA) in the brain have been suggested to play a role. Nevertheless, inhibition of neither MAO-A nor MAO-B influences the equilibrium of central dopamine levels: only when both isoenzymes are inhibited does dopamine expression increase. 2-PEA, which has a similar physiological activity to amphetamine in its potential to release dopamine, has been regarded to be the internal amphetamine [123]. Its brain levels increase significantly following MAO-B inhibition, and the activity of aromatic l-amino acid oxidase is also enhanced [124]. It has been proposed that these effects might contribute to the evident neuroprotective effects of MAO-B inhibitors. However, moclobemide, a selective reversible MAO-A inhibitor, has been indicated to possess anti-parkinsonian activity [125]. Clinical trials with selegiline in patients with Alzheimer’s disease have not given definite outcomes, though MAO-B expression is enhanced in the brains of such cases and might increase oxidative stress in this disease [32]. 

Moreover, it has been suggested that multitherapy with a MAO-B inhibitor and one of the clinically used cholinesterase inhibitors, such as physostigmine, might be advantageous. A corresponding technique is to merge these 2 enzyme inhibitory functions in the same molecule. For example, the drug ladostigil combines the pharmacophore of rasagiline and a carbamate cholinesterase inhibitory moiety. Its therapeutic actions involve neuroprotection, butyrylcholinesterase and acetylcholinesterase inhibition, and brain-selective MAOA/B inhibition [126]. It seems to be potential in increasing cognition in humans, as well as having antidepressant and anxiolytic activity [127] and is in clinical trials for Alzheimer’s disease at present. Other neurodegenerative diseases such as Huntington’s disease, schizophrenia, and amyotrophic lateral sclerosis (ALS) share multiple pathological characteristics with Parkinson’s disease and Alzheimer’s disease, like oxidative stress, iron accumulation, excitotoxicity, neuroinflammation, and the misfolding of toxic proteins that cannot be degraded after ubiquitination. Selegiline treatment has not proved to be beneficial in the therapy of ALS. Although, rasagiline and CGP 3466 have been reported to be efficacious in animal models of ALS [128]. A single-patient study has reported successful use of selegiline, in combination with the serotonin reuptake inhibitor fluoxetine, in Huntington’s disease [18].

## 6. Future Perspectives 

Investigations on MAO and its inhibitors have devoted considerably to our comprehension of aminergic neurotransmission. They contributed to the literature suggesting that MAO plays a crucial role in normal brain physiology, and its inhibitors have established a pivotal place in depression and parkinsonism drug therapy. Moreover, they have delivered extra momentum in the advancement of several enzyme inhibitors as therapeutic targets in the treatment of Alzheimer’s disease and Parkinson’s disease, along with the combination of distinct inhibitory effects in a solitary moiety. Although, there is still not a complete understanding of their neuroprotective functions, neither is it indicated whether these drugs are adequately efficacious in the aged individual to merit their long-term use in the treatment of neurodegenerative disorders. 

As previously mentioned, this unpredictability is correlated with limitations in the conduction of experimental studies, possibly aggravated by a bit random dosage preference. Though, the long-term safety of selegiline has now been well-established. Some MAO inhibitors and structurally related compounds are “in the pipeline” and are the subject of various clinical/preclinical studies. Among them, the propargyl amines can be mentioned (i.e., rasagiline which has been observed to be useful in an experimental model of amyotrophic lateral sclerosis); as well, it could be used in the treatment of multiple neurological diseases.

A range of aliphatic propargyl amines has also been proven to be potent neuroprotective compounds in various in vivo and in vitro animal models. The aldehyde-sequestering functions of phenelzine propose that several congeners of this compound should be scrutinized as possible neuroprotective drugs. By altering the sequence of an alkyl chain, the GABA-inhibiting action (MAO-inhibiting effect) of phenelzine can be adjusted while still preserving the aldehyde-sequestering activity. 

Preclinical research concerning the structure-activity relationships might then be directed to govern the respective significance of sequestering reactive aldehydes. The newly discovered MAOIs were found efficacious and less toxic, though would not be able to locate a potent and specific MAOI that can selectively be used in the treatment of AD. Uncovering the crystal structures of MAO isoforms has largely supported the comprehension of drug-receptor interactions at the molecular level. The ligand-based rational-drug design of specific MAOIs has intensified the synthesis of novel leads. Consequently, from now onwards the synthesis of potent MAOIs via rational-drug-design approaches and the development of novel MAOIs may assist to acknowledge drug-receptor interaction and develop potential therapeutic compounds in the treatment of AD. 

Various newly developed MAOI compounds could be further expanded to decrease their probable adverse effects as promised candidates for AD therapy. Computational and synthetic chemistry are the methods for synthesizing ligands with potential MAO inhibiting properties. In the future, it is considered that evaluated and assembled data in this review will accelerate objectives for designing novel MAOIs in the treatment of AD.

## 7. Conclusions

Neurodegenerative disorders, mainly Alzheimer’s disease (AD), imply an immense economical and psychological load on humankind all over the globe. Age-linked AD is catalyzed through multiple factors, one of which is the overactive MAO enzyme, which metabolizes several amine neurotransmitters and generates several neurotoxic end-products such as reactive aldehydes, hydrogen peroxide, thus contributing to the development of AD. Hence, MAO was considered as a requisite drug target in the treatment of AD and its inhibitors have largely granted our understanding of aminergic neurotransmission. The MAOIs persist to be under substantial attention and the concern of considerable exploration. Many of them may prove to be beneficial in the treatment of some neurodegenerative diseases, ischemic stroke, and drug abuse, either single or as supplementary drugs. In actual fact, their multi-sided actions could be a privilege, providing them suitability to be used in the treatment of various disorders. Studies to date have reported that the neuroprotective effects of these drugs are composite and, in some cases are independent of MAO inhibition. They continue to be useful pharmacological agents that have done great to enhance our understanding of pathways complexed with neuroprotection and have stated major indications for future progression of neuroprotective agents.

## Figures and Tables

**Table 1:** Pre-clinical and clinical evidence suggesting monoamine oxidase association with progression of Alzheimer’s disease.

Experimental Model	Controls	Stimulus/Drugs Employed	Method of Determination	Result/Conclusion	Ref.
11 autopsied brains of AD individuals	5 non-AD individuals	Lazabemide (MAO-B inhibitor) Ro41-1049 (MAO-A inhibitor)	Enzyme radiography	The concentration of MAO enzyme in the parietal cortex of AD was substantially higher as compared to controls.	[46]
3 autopsied brains of AD patients	3 autopsied brains of controls	L-deprenyl	Cryo-microtomy (autoradiography)	The AD patients had higher MAO-B expression than the controls	[47]
Plaque-associated astrocytes of brains of AD patients	Literature data	L-deprenyl, Pargyline, Iproniazid	Enzymatic assay	Increased MAO expression in brains of AD pIC_50_ value was determined of all drugs	[48]
Postmortem brains of AD	Autopsied brains of non-AD individuals	Gene silencing (sense and anti-sense siRNA)	PLA (in situ proximity ligation assay) Immunocytochemistry.	Immunocytochemistry revealed MAO-B staining in the frontal cortex, hippocampus, and entorhinal cortex whose intensity is higher in AD brains than in controls.	[49]
60 autopsied brains of AD patients	60 autopsied brains of controls	Apo-E4 status	HPLC (high-performance liquid chromatography) and immunodetection (SDS-PAGE)	Increased MAO-A activity in AD brains has been associated with prodromal and co-morbid neuropsychiatric symptoms and with neurodegeneration	[50]
APP/PS1 mice	Control mice (wild type)	Selegiline (10 mg/kg per day for 4 weeks)	Morris water maze test	The aberrant GABA level in APP/PS1 mice was significantly decreased to the levels of GABA observed in wild type control mice following selegiline treatment	[51]
Adult male Wistar rats with AD	Male Wistar rats as controls	Sembragiline (0.3%)	HPLC + ion-spray tandem mass spectrometry	Administration of sembragiline resulted in substantially decreased levels of ROS and prevented reduced dopaminergic neuron numbers in substantia nigra, when compared with vehicle-treated mice	[52]
Mouse model of AD	Control mice	2-photon MAO probe	Fluorescence TPM imaging	In vivo correlation between MAO and progression of AD-indicating MAO as a potential biomarker of AD.	[44]

**Table 2:** Monoamine oxidase inhibitors in the preclinical trials for the treatment of Alzheimer’s disease.

Drug	Category of the Drug	Mechanism of Action	Clinical Phase	Ref.
Ladostigil	Inhibitor of monoamine oxidases A and B	Prevention of age-related glial activation and spatial memory deficits Improvement of cognitive performance Modulation of amyloid precursor protein processing Upregulation of antioxidant activity and mRNA expression of antioxidant enzymes.	Phase 2	[111]
Selegiline	Selective and irreversible inhibitor of monoamine oxidase B	Regulation of cleavage of amyloid precursor protein Activation of phosphokinase C Inhibits amyloid-beta plaque formation antioxidant.	Phase 3	[42,112]
Rasagiline	Irreversible inhibitor of monoamine oxidase B	Modulates amyloid precursor protein and amyloid-beta processing Iron chelator Regulates the cell cycle	Phase 2	[113]
M-30	Inhibitor of monoamine oxidases A and B Iron chelator	Regulates proteolytic processing of amyloid precursor protein Iron chelator Reduces neuronal death and apoptotic DNA damage	Preclinical and clinical phase	[114]
